# Supplementary material for: Circulating hypervirulent Marek’s disease viruses in vaccinated chicken flocks in Taiwan by genetic analysis of meq oncogene
Source: PLoS One. 2024 May 10;19(5):e0303371. doi: 10.1371/journal.pone.0303371 (PMC11086920; doi:10.1371/journal.pone.0303371)
Supplement: S1 Table — (DOCX) [file pone.0303371.s001.docx]

**S1 Table Profile list of MDV-1 strains used in this study.**

| Strain | Pathotype | Country | GenBank Accession No. |
| --- | --- | --- | --- |
| TW/008/18 | NA^a^ | Taiwan | OQ576796 |
| TW/009/18 | NA | Taiwan | OQ576797 |
| TW/011/18 | NA | Taiwan | OQ576798 |
| TW/014/18 | NA | Taiwan | OQ576799 |
| TW/023/18 | NA | Taiwan | OQ576800 |
| TW/109/19 | NA | Taiwan | OQ576801 |
| TW/123/19 | NA | Taiwan | OQ576802 |
| TW/133/19 | NA | Taiwan | OQ576803 |
| TW/141A/19 | NA | Taiwan | OQ576804 |
| TW/141B/19 | NA | Taiwan | OQ576805 |
| TW/146/19 | NA | Taiwan | OQ576806 |
| TW/147/19 | NA | Taiwan | OQ576807 |
| TW/148/19 | NA | Taiwan | OQ576808 |
| TW/149/19 | NA | Taiwan | OQ576809 |
| TW/003/20 | NA | Taiwan | OQ576810 |
| TW/0229/20 | NA | Taiwan | OQ576811 |
| TW/048/20 | NA | Taiwan | OQ576812 |
| TW/116/20 | NA | Taiwan | OQ576813 |
| Tokachi-p1 | NA | Japan | AB638843 |
| Tokachi-s1 | NA | Japan | AB638844 |
| Tokachi-m1 | NA | Japan | AB638841 |
| OkiH26070 | NA | Japan | LC137001 |
| OkiH26035 | NA | Japan | LC137000 |
| Tn-n1 | vv | India | HM749324 |
| Tn-n2 | vv | India | HM749325 |
| TH/CBI/440/18 | NA | Thailand | ON931289 |
| TH/CBI/492/19 | NA | Thailand | ON931267 |
| TH/CCO/575/20 | NA | Thailand | ON931290 |
| TH/CBI/656/21 | NA | Thailand | ON931291 |
| MPF57 | v | Australia | EF523771 |
| 04CRE | v | Australia | EF523772 |
| 02LAR | vv | Australia | EF523773 |
| FT158 | vv | Australia | EF523774 |
| Woodlands1 | vv | Australia | EF523775 |
| GX070079 | vv | China | EU427304 |
| GX0101 | vv | China | JX844666 |
| GX070060 | vv | China | EU427303 |
| WS04 | vv | China | HQ638153 |
| WS03 | vv | China | HQ638152 |
| LS | vv | China | HQ638149 |
| LMS | vv | China | JQ314003 |
| SD2012-1 | vv+ | China | KC511815 |
| LTS | vv+ | China | KP888838 |
| GA | v | USA | AF147806 |
| 617A | vv | USA | AY362712 |
| Md5 | vv | USA | AF243438 |
| RB1B | vv | USA | EF523390 |
| 584A | vv+ | USA | DQ534532 |
| 648A | vv+ | USA | AY362725 |
| N | vv+ | USA | AY362719 |
| TK | vv+ | USA | AY362721 |
| 814 | vaccine | China | GU354326 |
| CU-2 | mild | USA | EU499381 |
| CVI-988 | vaccine | Netherland | DQ530348 |
| 3004 | vaccine | Russia | EU032468 |

^a^ NA: not available
