# Supplementary material for: Circulating hypervirulent Marek’s disease viruses in vaccinated chicken flocks in Taiwan by genetic analysis of meq oncogene
Source: PLoS One. 2024 May 10;19(5):e0303371. doi: 10.1371/journal.pone.0303371 (PMC11086920; doi:10.1371/journal.pone.0303371)
Supplement: Attachment — Submitted filename: Response to Reviewers.docx [file pone.0303371.s003.docx]

**Response to Reviewers**

Date: 04/14/2024

Manuscript ID: PONE-D-23-40205

Title: Circulating hypervirulent Marek’s disease viruses in vaccinated chicken flocks in Taiwan by genetic analysis of *meq* oncogene

Corresponding Author: Dr. Ming-Chu Cheng

Dear Chief Editor,

We thank for the opportunity to submit our revised draft of the manuscript titled “Circulating hypervirulent Marek’s disease viruses in vaccinated chicken flocks in Taiwan by genetic analysis of *meq* oncogene” for the publication in *PLoS One* journal. We appreciated the time and efforts that you and the reviewers devote to providing feedback about the manuscript along with insightful comments and valuable improvements to this study. We have incorporated most of the suggestions raised by the reviewers, and the revisions are highlighted within the revised manuscript. The point-by-point response, labeled in blue, to reviewers’ comments and concerns were listed below. Please refer to the describing line numbers in the revised manuscript file with tracked changes.

**Review Comments to the Author:**

**Reviewer #1**

1. Did the authors check the co-infection with ALV and REV in these cases? If yes, they can give the status of these oncogenic co-infections with MD, as in many cases, co-infection of these two predisposes the MD outbreak.

**Author responses:** In this study, ALV and REV were also detected by PCR, but no positive results were observed (described in Materials and Methods, “Line 96 “ and Results, “ Line 152-153, 157”).

2. Authors should have studied other oncogenes such as pp38, vIL-8 and PP38 also, why these oncogenes are not investigated as this sequence information would be useful

**Author responses:**

The high mutation rates and virulence-associated variations on *meq* gene and protein sequences have resulted in the construction of *meq* genes bank, which can provide abundant molecular information for epidemiological research on Marek’s disease virus. The *meq* gene has become most popular marker preferentially used for phylogenies and virulence identification of domestic MDV-1 strains by many research groups. Therefore, *meq* gene was chosen and analyzed in this study to reveal the spread of hypervirulent MDV-1 strains among chicken flocks in Taiwan. Other oncogenes, such as pp38, vIL-8 and PP38, were not investigated.

3. No isolation was done in this work, so authors should mention as MDV strains rather than isolates in their manuscript.

**Author responses:** Thank you for your suggestions. “isolates” were replaced by “strains” in the manuscript.

4. Also, how 18 MDV were obtained from 17 cases?

**Author responses:** Among the 18 MDV-1 strains, 2 MDV-1 strains (TW/141A/19 and TW/141B/19) were obtained from one case (Flock ID: P108-141 in Table1). More information were added into “Results” section (Line 147-150).

**Reviewer #2**

1. English language Writing of the manuscript must be improved before publication.

**Author responses:** The English writing has been improved and revised by American Journal Experts (AJE) before submission.

2. Results section could be improved and more detailed supporting evidences can be provided to support the conclusions. Would be great if the authors could evaluate the isolates in vivo to support their hypothesis.

**Author responses:** Some of the strains were isolated, and the animal challenging experiments will be arranged to determine the virulence in animals. Hopefully, the results would be presented in the future.

Sincerely,

**Ming-Chu Cheng**

Department of Veterinary Medicine, College of Veterinary Medicine, National Pingtung University of Science and Technology, 1, Shuefu Road, Neipu, Pingtung 91201, Taiwan

E-mail: mccheng@mail.npust.edu.tw, Tel.: +886-8-770-3202 ext. 5075.
